# Supplementary material for: Chryseobacterium herbae Isolated from the Rhizospheric Soil of Pyrola calliantha H. Andres in Segrila Mountain on the Tibetan Plateau
Source: Microorganisms. 2023 Aug 5;11(8):2017. doi: 10.3390/microorganisms11082017 (PMC10459008; doi:10.3390/microorganisms11082017)
Supplement: Supplementary file 1 [file microorganisms-11-02017-s001.zip › Supplementary Materials.pdf]

Figure S1. Transmission electron micrograph of cell of strain *herbae* pc1-10^{T}. Bar, 500 nm. The strain was incubated on R2A medium at 30°C for 48 h.

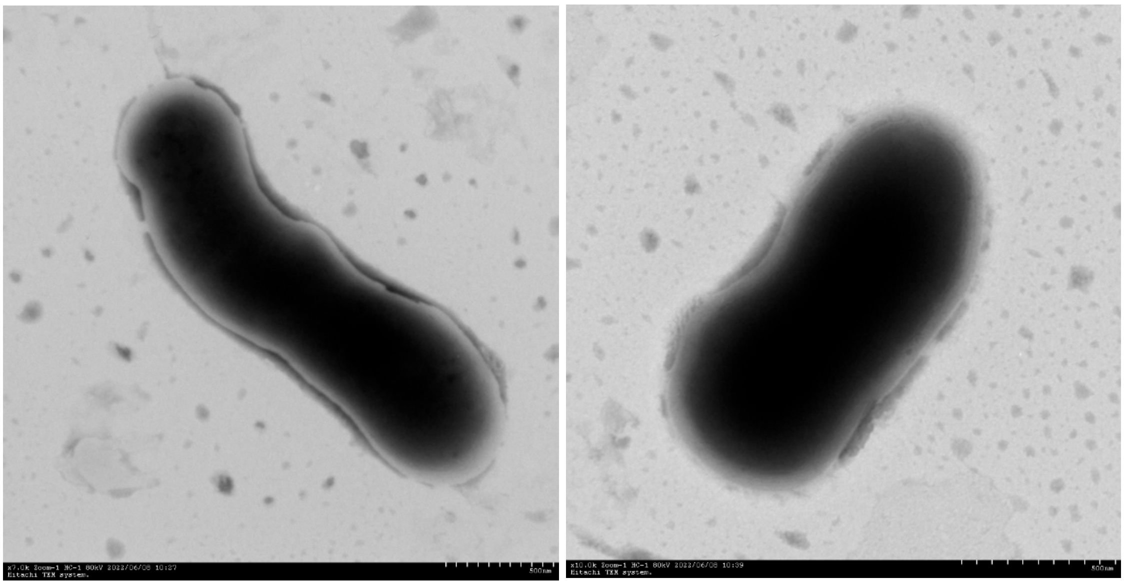

Figure S2. The polar lipids of strain *herbae* pc1-10^{T}. Polar lipids profiles of strains separated by two-dimensional TLC, which were detected by spraying with molybdatophosphoric acid reagent. (1), Total polarity; (2), Amino lipids; (3), Glycolipids; (4), phospholipids. PE, phosphatidethanolamine; AL1–5, aminolipids; L1–2, unknown lipids; GL1-3, glycolipids. All data are from this study.

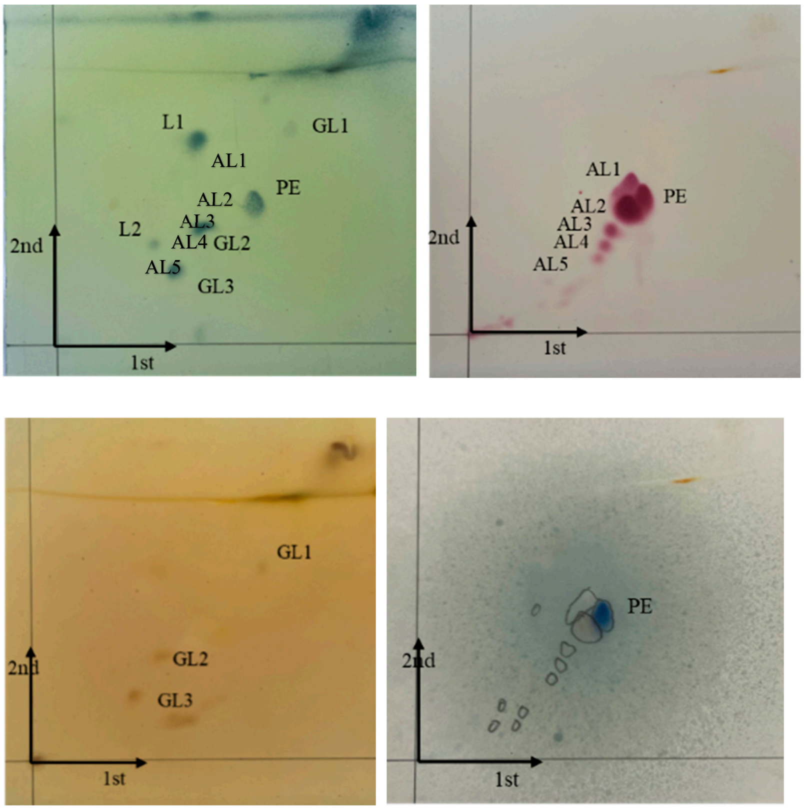

Figure S3. Maximum-likelihood phylogenetic tree of strain *herbae* pc1-10^T and its relatives based on the comparison of the 16S rRNA gene sequences. Genbank accession numbers were given in parentheses.

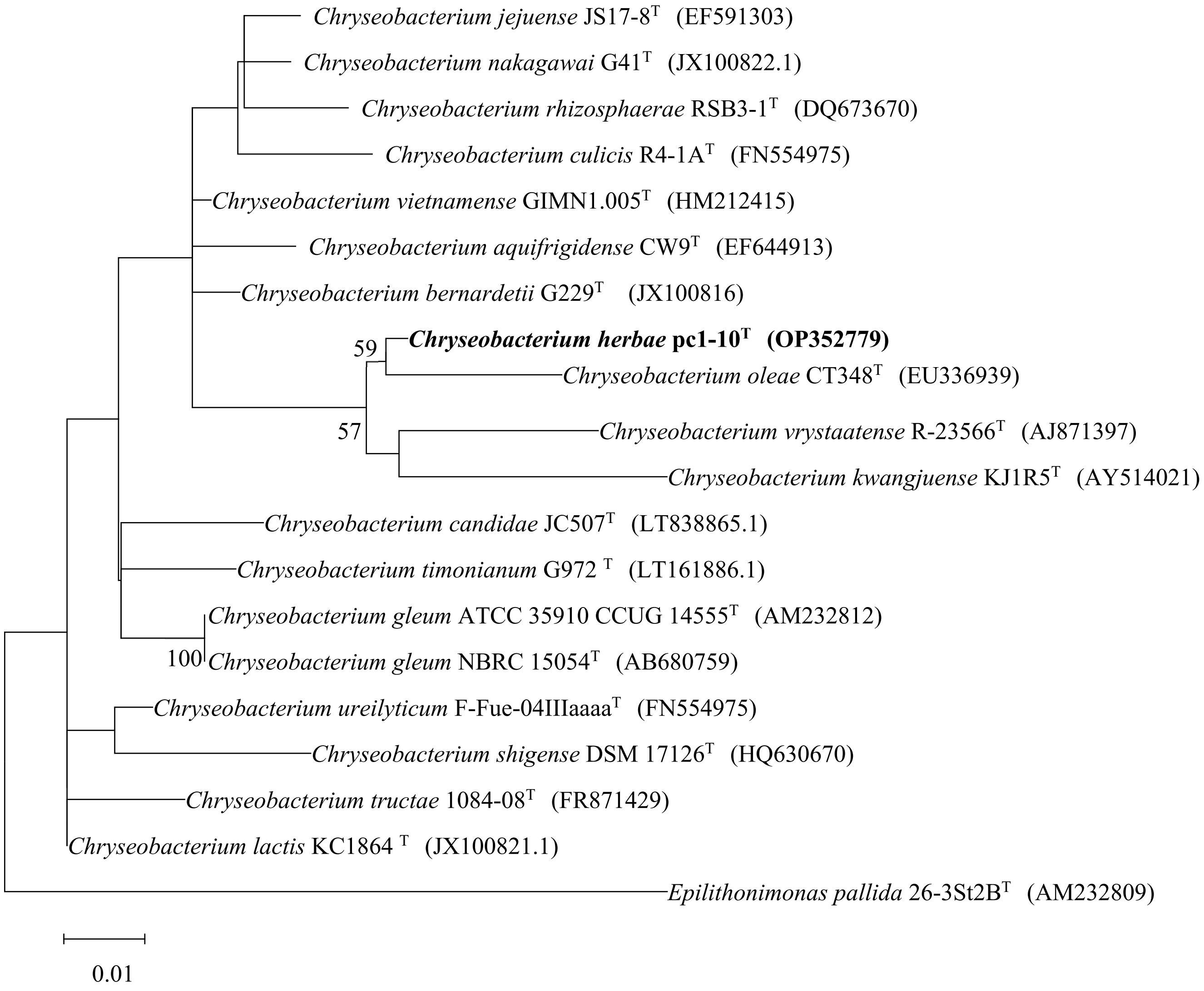

Figure S4. Growth morphology of strains on NA plates.

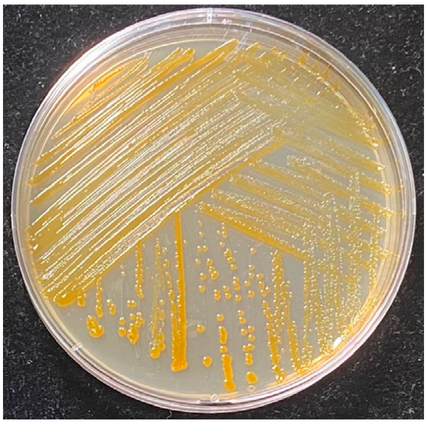

Figure S5. The indole acetic acid (IAA) standard (5–40 mg/L) curve.

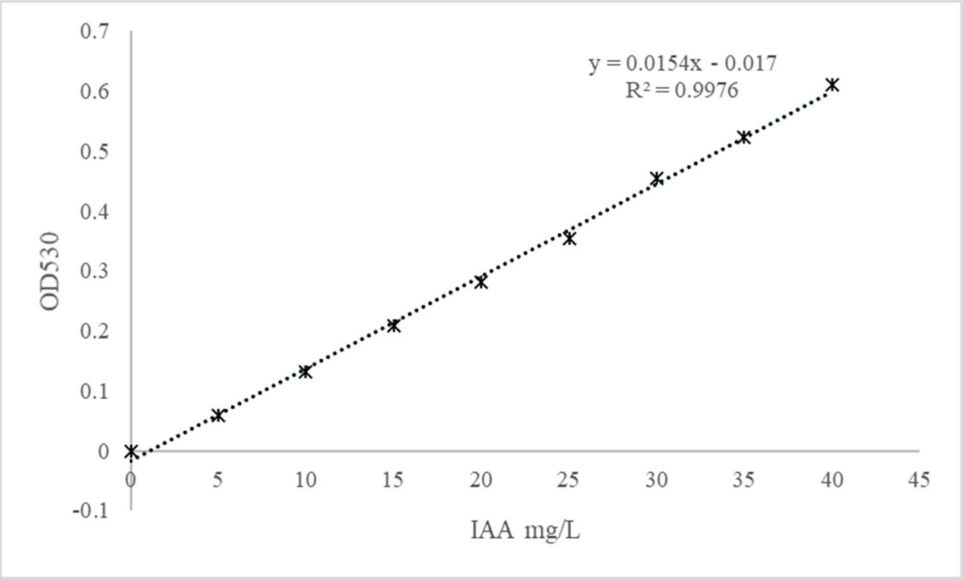

Table S1. The standard curve raw data and OD_{530} values of the strain *herbae* pc1-10^T to be tested.

IAA mg/L	0	5	10	15	20	25	30	35	40	pc1-10^T
OD_{530}	0.000	0.069	0.137	0.204	0.281	0.364	0.463	0.519	0.615	0.039
OD_{530}	0.000	0.047	0.136	0.220	0.280	0.360	0.452	0.520	0.608	0.038
OD_{530}	0.000	0.063	0.119	0.199	0.283	0.340	0.450	0.527	0.607	0.035

Figure S6. Antagonistic experiment of against pathogenic fungi (*Magnaporthe oryzae*) of rice blast.

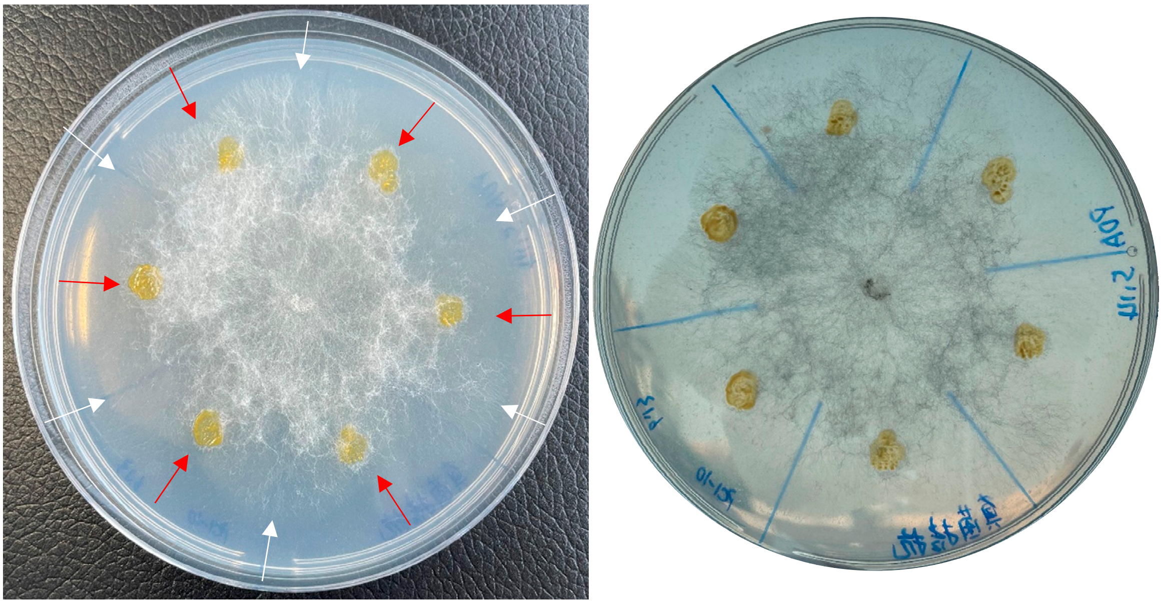

Notes: The strain *herbae* pc1-10^T slows down fungal hyphal growth.

Figure S7. Reversible colour change of pigment of strain *herbae* pc1-10^T

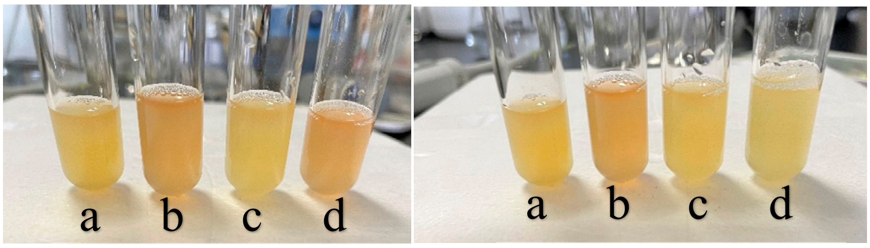

Notes: control culture (a); colour changes to red when 3 % KOH is added to the broth culture (b); colour changes from red to yellow when 3 % KOH is added first, followed by 1.5 N HCl (c); colour changes from red to yellow when 3 % KOH is added first (left d), followed by 1.5 N HCl (right d)

16S rRNA gene sequence of *Chryseobacterium herbae sp.nov.* (from genome)
>Chryseobacterium *herbae* pc1-10^T (OP352779)
AGGCTTACCTAGGCAGCTCCTATTACGGTCACCGACTTCAGGTACCCCAGACTTCCATGGCT
TGACGGGCGGTGTGTACAAGGCCCGGGAACGTATTCACCGCGCCATGGCTGATGCGCGATT
ACTAGCGATTCCAGCTTCATAGAGTCGAGTTGCAGACTCCAATCCGAACTGAGACCAGCTT
TCGAGATTCGCATCCAGTCACCTGGTAGCTGCCCTCTGTACTGGCCATTGTATTACGTGTGT
GGCCCAAGGCGTAAGGGCCGTGATGATTTGACGTCATCCCCACCTTCCTCTCTACTTGCGTA
GGCAGTCTCACTAGAGTCCCCAACTTAATGATGGCAACTAGTGACAGGGGTTGCGCTCGTT
GCAGGACTTAACCTAACACCTCACGGCACGAGCTGACGACAACCATGCAGCACCTTGAAA
AATGTCCGAAGAAAAGTCTATTTCTAAACCTGTCATTTCCCATTTAAGCCTTGGTAAGGTTC
CTCGCGTATCATCGAATTAAACCACATAATCCACCGCTTGTGCGGGCCCCCGTCAATTCCTTT
GAGTTTCAGACTTGCGTCCGTACTCCCCAGGTGGCTAACTTATCACTTTCGCTTAGTCTCTG
AATCCGAAAACCCAAAAACGAGTTAGCATCGTTTACGGCGTGGACTACCAGGGTATCTAAT
CCTGTTCGCTCCCCACGCTTTCGTCCATCAGCGTCAGTTGTTGCTTAGTAACCTGCCTTCGC
AATTGGTGTTCTAAGTAATATCTATGCATTTCACCGCTACACTACTTATTCCAGCTACTTCAAC
AACACTCAAGACTTGCAGTATCAATGGCAGTTTCACAGTTGAGCTGTGAGATTTCACCACT
GACTTACAAATCCGCCTACGGACCCTTTAAACCCAATAAATCCGGATAACGCTTGCACCCTC
CGTATTACCGCGGCTGCTGGCACGGAGTTAGCCGGTGCTTATTCGTATAGTACCTTCAGCTA
GATACACGTATCTAGGTTTATCCCTATACAAAAGAAGTTTACAACCCATAGGGCCGTCGTCCT
TCACGCGGGATGGCTGGATCAGGCTCTCACCCATTGTCCAATATTCCTCACTGCTGCCTCCC
GTAGGAGTCTGGTCCGTGTCTCAGTACCAGTGTGGGGGATCACCCTCTCAGGCCCCCTAAA
GATCGTAGACTTGGTGAGCCGTTACCTCACCAACTATCTAATCTTGCGCGTGCCCATCTTTAT
CCACCGGAGTTTTCAATATCAAGTGATGCCACTTAATATATTATGGGGTATTAATCTTCCTTTC
GAAAGGCTATCCCCCTGATAAAGGCAGGTTGCACACGTGTTCCGCACCCGTGCG

Table S2. Statistics of protein identification results

Name	Total spectra	Matched spectrum	Peptide	Identified protein
Cell	78,582	42,645	19,568	2,455
Supernatant	79,597	52,846	23,815	2,650
